# Channing on Etherization in Child Birth

**Published:** 1849-05

**Authors:** 


*Channing on Etherization in Child Birth.*—Inquiries having been frequently addressed to us respecting this new work, we would inform those desirous of obtaining it, that a supply of copies has been obtained by G. H. Derby & Co., booksellers of this city, who will be happy to answer orders for this, and other Medical books, promptly.

